# LipidHome: A Database of Theoretical Lipids Optimized for High Throughput Mass Spectrometry Lipidomics

**DOI:** 10.1371/journal.pone.0061951

**Published:** 2013-05-07

**Authors:** Joseph M. Foster, Pablo Moreno, Antonio Fabregat, Henning Hermjakob, Christoph Steinbeck, Rolf Apweiler, Michael J. O. Wakelam, Juan Antonio Vizcaíno

**Affiliations:** 1 EMBL Outstation, European Bioinformatics Institute, Wellcome Trust Genome Campus, Hinxton, Cambridge, United Kingdom; 2 The Babraham Institute, Babraham Research Campus, Cambridge, United Kingdom; Governmental Technical Research Centre of Finland, Finland

## Abstract

Protein sequence databases are the pillar upon which modern proteomics is supported, representing a stable reference space of predicted and validated proteins. One example of such resources is UniProt, enriched with both expertly curated and automatic annotations. Taken largely for granted, similar mature resources such as UniProt are not available yet in some other “omics” fields, lipidomics being one of them. While having a seasoned community of wet lab scientists, lipidomics lies significantly behind proteomics in the adoption of data standards and other core bioinformatics concepts. This work aims to reduce the gap by developing an equivalent resource to UniProt called ‘LipidHome’, providing theoretically generated lipid molecules and useful metadata. Using the ‘FASTLipid’ Java library, a database was populated with theoretical lipids, generated from a set of community agreed upon chemical bounds. In parallel, a web application was developed to present the information and provide computational access *via* a web service. Designed specifically to accommodate high throughput mass spectrometry based approaches, lipids are organised into a hierarchy that reflects the variety in the structural resolution of lipid identifications. Additionally, cross-references to other lipid related resources and papers that cite specific lipids were used to annotate lipid records. The web application encompasses a browser for viewing lipid records and a ‘tools’ section where an MS1 search engine is currently implemented. LipidHome can be accessed at http://www.ebi.ac.uk/apweiler-srv/lipidhome.

## Introduction

A key area of metabolomics research is performed in the field of lipidomics, which deals with the detection and identification of lipids, fatty acids and derivatives. Lipids are a broad class of molecules with a variety of structures. They have wide ranging functions across the entire breadth of biological kingdoms, including roles in the structure of cell membranes, energy storage and cell signalling [1]. Perturbations to the lipidome have been reported in several human diseases such as diabetes [1], obesity [1], Alzheimer’s disease [2], liver disease [3], hypertension [4] and schizophrenia [5]. The workhorse of high throughput lipidomics is mass spectrometry (MS), often generating hundreds of lipid identifications in a single experiment. The first high throughput lipidomics MS publications emerged in 1994 [6], [7] and have since gained considerable momentum with dozens of papers published *per* year. This is partially due to several reasons such as the technical improvements in the mass spectrometers and the improved availability of reagents and synthetic standards necessary for quantitative lipidomics.

Central to all “omics” disciplines including lipidomics is the high-throughput identification of biological molecules followed by their accurate description and reporting. Key to this type of research is the existence of a centralized namespace against which to identify the molecules. Fields such as proteomics have a long history of providing databases of proteins, with standardised naming formats and intelligent mappings that relate protein names in different resources. Possibly the prime example of this is the UniProt resource [8], [9], self-described as “a comprehensive resource for protein sequence and annotation data”. It provides not only protein sequence information for a multitude of organisms but a considerable amount of metadata for those sequences, ranging from functional and structural to disease related annotations and supporting evidence in the literature.

In the lipidomics field, there are several publicly available reference databases of lipids. Among them, the most prominent resources are LipidDAT [10], LipidBank [11] and perhaps most importantly, the LIPID MAPS Structural Database (LMSD) [12]. Even LMSD, usually regarded as the ‘gold standard’ for referencing lipid identifications, lacks some typical features of a reference database. For instance, the availability of cross-references to the literature and a transparent curation protocol, as well as support for some common lipidomics specific use cases.

**Figure 1:**
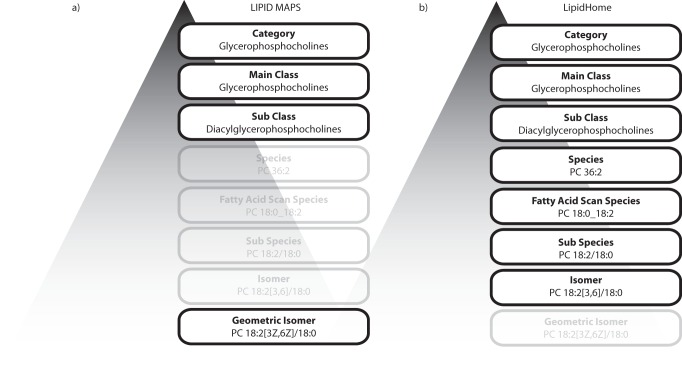
The structural hierarchy of lipid records. **a)** *The structural hierarchy of lipid records in the Lipid Maps Structural Database (LMSD)*. The Lipid Maps classification system organises all lipids into “*Categories*”, “*Main Classes*” and “*Sub Classes*”. The lipid records are stored at the “*Geometric Isomer*” level where the total number of carbons, total number of double bonds, the position of double bonds and the stereochemistry of double bonds are defined for each fatty acid. The transparent lipid identification hierarchy levels are not supported by the LMSD. **b)** *The structural hierarchy of lipid records in the LipidHome database*. Similar to the LIPID MAPS classification system, lipids are organised into “*Categories*”, “*Main Classes*” and “*Sub Classes*”. Lipid records are stored at four levels. Each level relates to a typical type of identification from a high throughput mass spectrometry experiment. From structurally undefined “*Species*” typically identified from a single precursor ion mass, to structurally resolved “*Isomer*” level identifications.

A common problem in the field of lipidomics is the mismatch between the lipid identifications reported in the literature and the MS-based evidence available to back up those identifications. The existing reference systems for lipid identifications store lipids usually at a very high structural resolution. As a consequence, there is a disparity between the structural resolution of data that the large majority of experimentalists in the field are producing and the reference records stored in the existing lipid databases. For instance, the LMSD structural hierarchy (**Figure 1a**) shows that the lipids can be stored as “*Geometric Isomers*”, where not only double bond positions are defined on the component fatty acids, but also the stereochemistry of those double bonds. In addition, considering the billions of physically feasible lipid “*Geometric Isomers*”, the LMSD has an extremely limited coverage of the chemical space at the structural resolution they support. Another existing issue in lipid databases is their inability to distinguish between the experimentally validated and the theoretically possible lipids. Again, in the case of the LMSD, lipid product ion mass spectra have recently been made available for a number of lipids but this information is not so obvious and easy to access. As a consequence, it is often assumed that all records are experimentally validated.

Alongside lipid databases there are an increasing number of tailored bioinformatics tools for a range of applications. For MS identification, LipidXplorer [13], MZmine [14] and Lipid Data Analyzer [15] are three well-documented software tools. However, the uptake of these tools is slow with many labs still identifying spectra by hand. The LIPID MAPS consortium also provides additional tools for structure drawing, pathways and a database of lipid related genes and proteins [16], [17].

In this work we describe the development and features of a new resource called ‘LipidHome’. The motivation for this work was to fill the gap between existing lipid databases and the current need to accurately reflect identifications generated by modern high throughput MS approaches. LipidHome provides stable identifiers to lipid identifications at all levels of lipid structural resolution currently unavailable in existing resources. In addition, it maintains a clear separation of the theoretically feasible *in silico* predicted lipids from the experimentally validated lipids. The final result is a scalable and robust bioinformatics platform including a database and a web application, that can be used for the referencing of lipid identifications and makes use of the latest developments in lipid nomenclature standardisation [18]. This functionality supports the increasing trend in different fields to provide accurate cross-referencing of experimental identifications to a stable namespace.

## Materials and Methods

### 1) Lipid Structural Identification Hierarchy

MS-derived lipid identifications are obtained at various levels of structural resolution (**Figure** S**1 in File** S**1)**. To understand how LipidHome is structured, it is first necessary to know how the lipids are hierarchically organized in the ‘LipidomicNet’ (http://www.lipidomicnet.org) standard lipid nomenclature [18], optimized for high-throughput MS-based lipidomics approaches. The supported lipid hierarchy levels are: “*Category*” “*Main Class*”, “*Sub Class*”, “*Species*”, “*Fatty Acid Scan Species*”, “*Sub Species*” and “*Isomers*”.

To summarize, the lipid “*Main Class*” is often inferred from the sample preparation protocol. From a single MS1 scan and single precursor ion mass (e.g. 759.58 Da), no structural resolution on the composite fatty acids is available, only the total number of carbons and double bonds of the lipid can be estimated (a “*Species*” level identification e.g. PC 34∶1 or PC O-35∶1). Performing an MS2 scan on the fragmented precursor ion generates a set of product ions capable of identifying the fatty acids, although the *sn* position of those fatty acids usually remains unknown unless the intensity can be appropriately taken into account with the instrument set up at hand [19]. This type of experiment gives rise to identifications at the “*Fatty Acid Scan Species*” (e.g. PC 16∶0_18∶1) and “*Sub Species*” (e.g. PC 18∶1/16∶0) levels, respectively. Using specialist protocols and complex downstream data analysis, it is not only possible to identify the composition of individual fatty acids but also the positions of the double bonds on their hydrocarbon chains [20], [21] (e.g. PC 18∶1 [9]/16∶0). However, this level of “*Isomer*” detail is rarely achieved in high throughput studies and at present it is limited to a few laboratories with highly specific research interests. As a consequence, it is scientifically inaccurate to report anything other than a “*Species*” level identification (e.g. PC 36∶2) from a single precursor ion mass. Unfortunately, the reporting of much higher structural resolution identifications from precursor ion masses is common in the literature (e.g. PC 18∶0/18∶2[3Z,6Z], for this case), and the lipidomics community must actively work to discourage the reporting of misleading results.

In LipidHome, in line with the recommendations from the LIPID MAPS consortium [22], lipids are organised into the same concept of “*Category*” and “*Main Class*” as found in the LMSD. The only minor alteration to the established classification system is at the “*Sub Class*” level. In the LMSD the linkage position specific variants are organised into two lipid sub classes (for example, “1-alkyl,2-acylglycerophosphocholines [GP0102]” and “1-acyl,2-alkylglycerophosphocholines [GP0108]). However, in LipidHome these two sub classes are collapsed into the single “*Sub Class*” “monoalkyl,monoacylglycerophosphocholines”, where linkage position is not implied. This alteration to classification more closely aligns the information available from lipid MS-based identifications with the LipidHome structural hierarchy, where an alkyl linkage may be identifiable by mass, but its *sn* position remain unknown.

### 2) LipidHome Database Content

As described previously, the LMSD stores the vast majority of its lipid identifications at the “*Geometric Isomer*” level, where double bond positions and stereochemistry are fixed. In LipidHome (**Figure 1b**), lipids are stored at all the relevant structural hierarchy levels. As a consequence, the LipidHome database schema reflects this structural hierarchy (**Figure S2 in File** S**1**). In the first release of LipidHome reported in this manuscript, theoretical lipids are generated for the “*Categories*” glycerolipids and glycerophospholipids (**Figure S3 in File** S**1)**. However, it is important to highlight that the general approach and methodology have been designed to be readily transferable to other lipid categories (with only minor modifications to the code). For example while the rules for steroid enumeration are not yet implemented, the rules engine that would handle them has already been done. Steroids represent a slightly more challenging enumeration process, but they still are governed by an albeit rather different set of rules: the four conjoined cycloalkane rings, a number of modified sites (treated the same as the *sn1*, *sn2* and *sn3* positions of glycerol) and a variable oxidation state of the rings (handled in the same manner as the double bond positioning on fatty acids).

As an example of the logic followed, the generation of glycerophospholipids is described here. This category of lipids is composed of distinct parts: head group, phosphate, glycerol, linkages and fatty acids (**Figure S4 in File** S**1**). By doing a Cartesian cross of all “*Main classes*” and fatty acid linkage combinations (“acyl” or “alkyl”), a set of lipid “*Sub Classes*” was generated. Within a particular lipid “*Sub Class*”, the variability is due to the fatty acid composition. Each of these “*Sub Classes*” has two fatty acid ‘R’ groups, which are filled by a generator of all theoretically possible fatty acids, bound by a set of chemical rules.

In order to estimate the chemical bounds for fatty acid generation a survey was conducted amongst members of the ‘LipidomicNet’ consortium. A set of parameters for fatty acid generation was agreed upon as:

- Minimum number of carbons: 2.
- Maximum number of carbons: 30.
- Odd number of carbon fatty acids: Fatty acid chains may have an odd number of carbons.
- Minimum number of double bonds: 0.
- Double bond spacing: Double bonds are constrained to appear at a ‘3*n*+2′ distance, where *n* is any non-negative integer which fits within the length of the fatty acid chain. Bonds can be skipped, but the distance between any two bonds needs to fulfil this condition, in line with the majority of lipid “*Geometric Isomers*” in the LMSD.
- First double bond: Can only appear at bond position 2 or higher.

While these parameters do not fully reflect the enormous variety of fatty acids synthesised in nature such as branch-chain fatty acids, they were used to construct a large proportion of the lipids of interest within the lipidomics research community.

### 3) Technical Implementation

**Figure 2:**
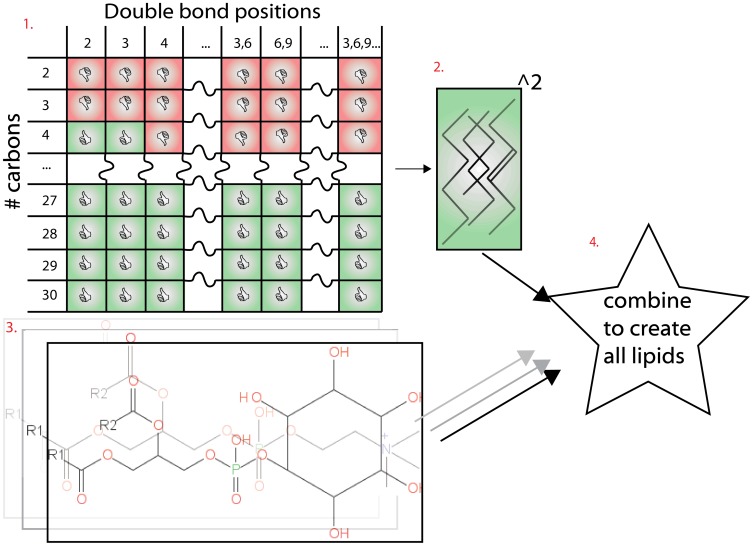
A diagram of in silico construction of theoretical diradyl lipid “Sub Species”. Steps: 1. All viable potential fatty acids are generated from a set of starting parameters; 2. They are combined all against all; 3. The head groups with alpha-carbons and linkages are generated; 4. The head groups are crossed with the fatty acid pairs to produce all viable lipid structures within the predefined chemical space.

The complete lipid generation process (**Figure 2**), was provided by the open source ‘FASTLipid’ Java library (https://github.com/pcm32/FASTLipid), which relies on the computational chemistry framework ‘Chemical Development Kit’ (CDK) [23]. ‘FASTLipid’ can be used as a standalone Java application for the generation of theoretical lipids, but also for local mass searches of ‘on-the-fly’ generated structures. ‘FASTLipid’ differs significantly in its approach to the generation of theoretical lipids, in comparison to the recently published ‘LipidMapsTools’ [24]. Whole lipids are not enumerated from a restricted set of “Fatty Acid Isomers” but from a set of flexible rules. The ‘FASTLipid’ library enumerates not just chains onto the phosphoglycerol body but also all possible double bond positions within the fatty acids, producing considerably more possible lipid “*Isomers*”. For example, ‘FASTLipid’ is capable of generating the “*Isomer*” PC 18∶1 [3]/16∶0 by defining the appropriate fatty acid structural components (chain 1∶18 carbons, 1 double bond at position 3, chain 2∶16 carbons, no double bonds). Attempting the generation of the same with ‘LipidMapsTools’ will fail because it has no predefined fatty acid 18∶1 [3].

Cross references to existing lipid databases were harvested primarily from the LIPID MAPS database. From each LIPID MAPS record its secondary cross references to other resources such as KEGG [25] and PubChem [26] were also extracted and stored in the LipidHome database. This was achieved with a set of scripts that utilised the LIPID MAPS web service (**Supplementary Note S1a in Supplementary File S1**) and annotated lipids at the “*Isomer*” level in the LipidHome database. In a second round of cross-referencing the Chemical Entities of Biological Interest (ChEBI) [27] web service was used to extract cross references at the “*Sub Species*” level (**Supplementary Note S1b in Supplementary File S1**). For the creation of cross-references to the literature, the LipidHome database was synonymised to a set of non-standard lipid nomenclatures in common use. Each record and its synonyms were searched against the entirety of MEDLINE abstracts using the “Whatizit” web service (http://www.ebi.ac.uk/webservices/whatizit/) [28]. The details of any citation found to contain a lipid name in its title, abstract or keywords were added to the database and referenced to the appropriate record (**Supplementary Note S1c in Supplementary File S1**).

The LipidHome web application is built on the SPRING application development framework (http://www.springsource.org/), using a *Model View Controller* approach. Data from the MySQL (http://www.mysql.com) database is relayed using a robust and flexible web service to the web application presentation layer, implemented using the ExtJS framework (http://www.sencha.com/products/extjs).

Lipid chemical structures are rendered using a modified version of the ChemDoodle JavaScript library of web components (http://code.google.com/p/lipidhome-molrender/). The source code is available to download from http://code.google.com/p/lipidhome/. The application is running on an Apache Tomcat (http://tomcat.apache.org/) web server at the European Bioinformatics Institute at http://www.ebi.ac.uk/apweiler-srv/lipidhome. For optimal user experience we recommend using the Google Chrome (https://www.google.com/chrome) web browser.

## Results

### 1) Population of the LipidHome Database

Once the rules to create the theoretical lipids were agreed, they were generated using the ‘FASTLipid’ library. The lipid “*Isomers*” were generalised to the lipids of lower structural resolution: “*Sub Species*”, “*Fatty Acid Scan Species*” and “*Species*” and subsequently persisted to a custom MySQL database (**Figure S2 in File** S**1**). The whole process generated 20,297 “*Species*”, and 36.15 million “*Sub Species*” for the 39 lipid “*Sub Classes*” described in **Figure S3 in File** S**1**.

In addition to storing lipid names, the corresponding lipid structure images were also stored. These images are available for download and follow the same rules for structural representation used by the ChEBI database [27]. The challenge of representing lipids at levels that are not structurally defined (such as “*Species*”, “*Fatty Acid Scan Species*”, “*Sub Species*”) had not been addressed before by any other lipid reference database. Thus, the focus on structurally unresolved lipid identifications required the establishment of rules for their visualisation. Lipids with unresolved fatty acids are displayed as containing “R1”, “R2” and “R3” chains, where ‘1′, ‘2′ and ‘3′ denote the *sn* position of the fatty acid. Fatty acids are not drawn on molecules unless the double bond positions are known, i.e. an “*Isomer*” level identification. For “*Sub Classes*” with non-symmetric fatty acid linkages, the non-acyl linkage is always shown in *sn* position 1.

In addition, lipid records within the LipidHome database were annotated with references to external resources that contained record specific information. The more integrated a resource is with others the better visibility it has to new users. Other resources effectively offer free information that can be linked to, at a cost much lower than regenerating the information they contain from scratch. As the LMSD represents the most widely used lipid resource, it was the primary source of cross-references for LipidHome. Since the LMSD stores lipid identifications at the “*Geometric Isomer*” level, the only cross-references extracted were located at the LipidHome “*Isomer*” structural hierarchy level. Furthermore, cross-references already provided by the LMSD to other resources such as KEGG [25] and PubChem [26] were also extracted. The cross referencing scripts generated 7,641 primary cross-references to LIPID MAPS and extracted 1,139 secondary cross-references to other resources. The ChEBI database [27] was also searched for lipid records and resulted in 114 cross-references, added at the “*Sub Species*” level.

The LipidHome database also contains cross-references to the literature. The addition of which was done by EBI text-mining service; ‘Whatizit’ [28]. The process identified 290 lipid citations in the literature which were subsequently added to the database. Known literature for specific lipids can now be accessed *via* LipidHome rather than attempting multiple searches with unfamiliar nomenclatures, using tools that only search an article’s title and keywords.

### 2) Description of the LipidHome Web Application

**Figure 3:**
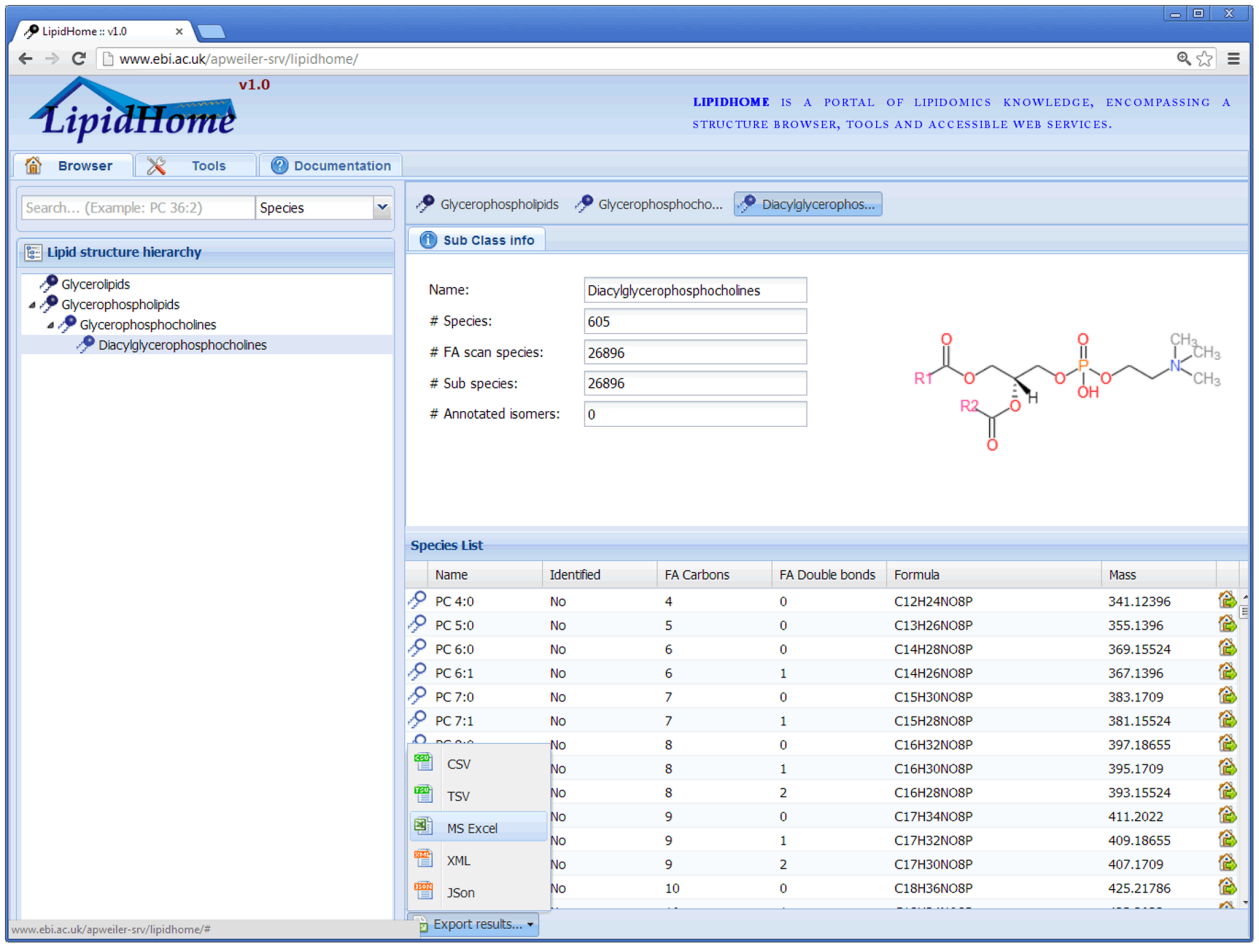
Screenshot of the LipidHome “Browser” view. The LipidHome structural hierarchy can be navigated in the far left tree panel. Clicking on a lipid record produces two vertically stacked panels in the right hand panel. The top panel shows general information about the selected record including an image. The bottom panel displays a table of the selected records’ children lipids, i.e. selecting the “*Sub Class*” “Diacylglycerophosphocholines” provides a list of its “*Species*”. These lists are exportable to a number of file formats.

The LipidHome web application is split into three main sections; “Browser”, “Tools” and “Documentation”. The “Browser” (**Figure 3**) is the core of the application where lipid records are navigated to and displayed. The interface is organised into two main panels. The left panel shows the lipid hierarchy tree: users can click the elements in this tree and they will be loaded into the right panel. Lipid records loaded into the right panel display information specific to the selected lipid in the top sub panel and a list of the record’s children in the bottom sub panel. For example selecting the “*Sub Class*” diacylglycerophosphocholines, will display an image and information about the “*Sub Class*” at the top and a list of its *“Species”* at the bottom. Records in the list can be selected to drill deeper down the structural identification hierarchy. Due to the large number of theoretical lipids stored in the LipidHome database, children of a selected lipid record (displayed in the table below the general information) are filterable by the table’s columns. As an example, the unidentified lipid records may be filtered out of the table by selecting the drop down menu of the “Identified” column header, hovering the mouse over “Filters” and selecting “Yes”. Alongside general information, cross-references and papers are available for some records (**Figure S5 in File** S**1**). From here links can be followed through to external resources with lipid specific information.

The web application is not the only way to access the information stored in the LipidHome database. Lipid records and a number of other views of the data are provided by a programming language independent web service (**Note S2 in File** S**1**). The web service allows the computational access of all the data views present by the web application, e.g. the list of a “*Species*” children, its “*Fatty Acid Scan Species*”.

**Figure 4:**
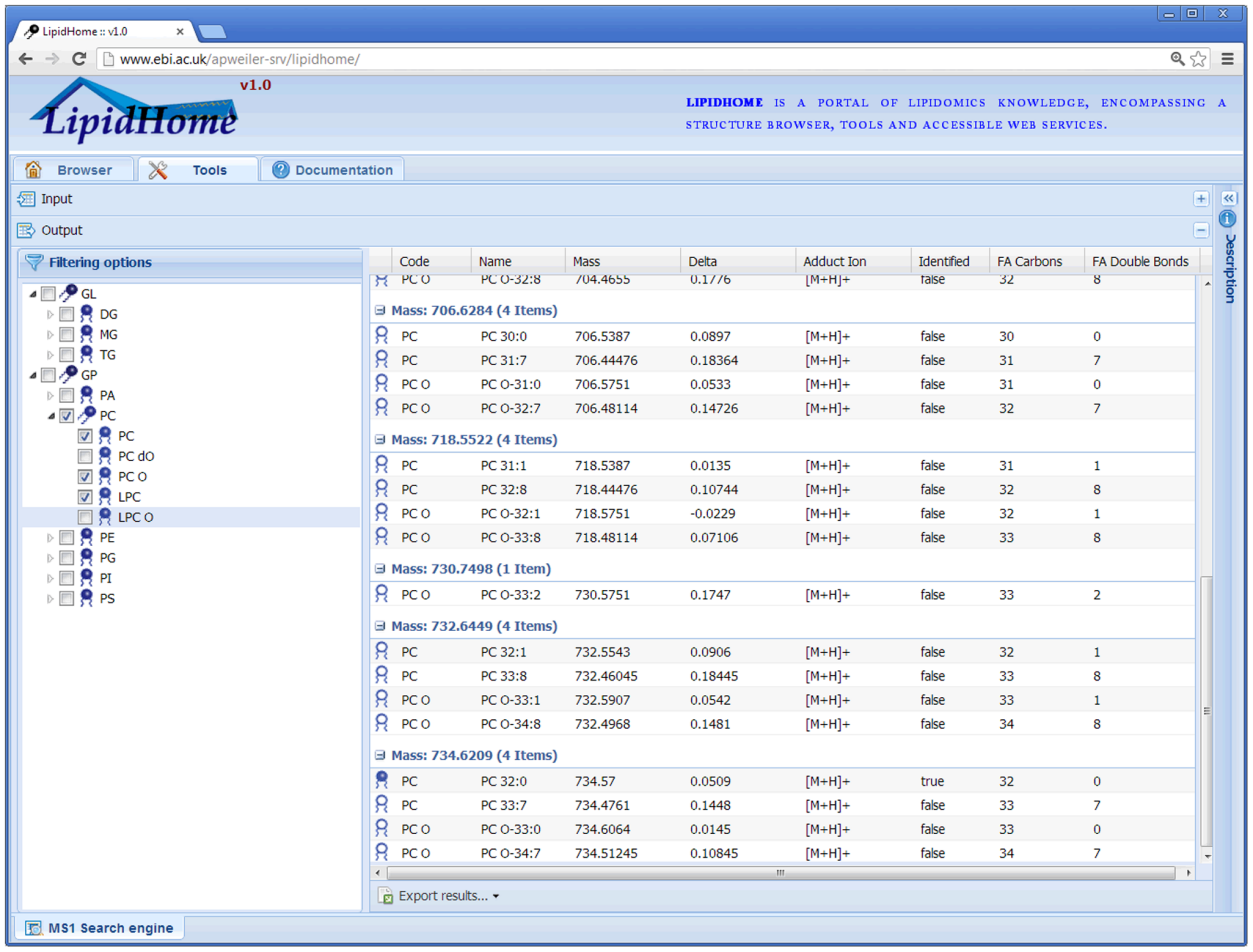
Screenshot of the output of an “MS1 search engine”. Results are split into each input search mass and their corresponding lipid “*Species*” identifications. The columns are filterable and sortable so that results may be organised prior to exporting them in a number of available file formats. In addition, whole lipid “*Categories*”, “*Main Classes*” and “*Sub Classes*” may be filtered out for the purpose of simplifying the results or replicating any step in the experimental protocol that may have isolated specific sets of lipids prior to the MS analysis.

As an example of the extensibility of LipidHome, an MS1 precursor ion search engine was implemented on top of the web services and embedded into the LipidHome web application. The search engine takes as inputs a “newline” separated list of precursor ion masses, mass tolerance and a set of adduct ions to pre-process the precursor ions with (**Figure S6 in File** S**1**). The processed masses are searched against “*Species*” in the database with the tolerance provided and hits returned in the output panel (**Figure 4**). Multiple adduct ions may be selected in a single search. However, increasing the search space will inevitably increase the number of acceptable isobaric lipid hits. Results can be filtered to exclude whole lipid “*Categories*”, “*Main Classes*” or “*Sub Classes*” in order to utilise any *a priori* knowledge of detected lipid species from e.g. solid phase extraction of single lipid “*Main Classes*”. Finally, results may be exported to a number of file formats for offline inspection and reporting. From a single precursor ion mass value only, it is not possible to distinguish the true identity of a search mass that hits multiple isobaric lipid species. It is the user’s responsibility to accurately and reproducibly report their results.

## Discussion

LipidHome provides the most comprehensive coverage of the glycerophospholipid and glycerolipid chemical space to date and is built upon an extensible rules based system for generating new theoretical lipids. Lipids are organised into a novel lipid identification structural hierarchy, enabling lipid identifications from high throughput MS based lipidomics to be accurately referenced to a stable namespace with community agreed upon nomenclature. Specifically, the field of lipidomics is producing lipid identifications most frequently from MS1 only experiments. These results can only accurately identify lipids at the “*Species*” level. The novel lipid structural hierarchy of LipidHome allows to exclusively provide a stable reference space for the majority share of high throughput lipidomics data currently being generated. Aside from its principal function as a dictionary of theoretical lipids, this functionality is enhanced with cross-references to validated lipid records in external databases. Furthermore, the entirety of MEDLINE was text mined for lipids at all levels of the lipid structural hierarchy, in order that records in LipidHome may be searched for and the abstracts of papers that mention them. Due to its limited scope and accuracy, text-mining approaches cannot substitute the human curation provided by resources like UniProt (in the subset UniProtKB/SwissProt). However, we believe these approaches are suitable when no feasible alternatives are currently available.

We expect that LipidHome and the associated MS1 search engine tool will be very useful for lipidomics scientists. For instance, a typical use case addressed by LipidHome allows for listing all the “*Fatty Acid Scan Species*” for a specific “*Species*”, and then to filter this list for lipids containing the fatty acid species (for instance “18∶1″). In addition, the new search engine implemented for MS1 precursor ions, allows the user to set the mass tolerance and expected adduct ions as search parameters. After filtering and sorting, the results can be exported to a number of formats for offline inspection and reporting. At the time of publishing, the equivalent tool from LIPID MAPS does not offer this functionality and as such results cannot be plugged into existing bioinformatics analysis pipelines.

LipidHome is built on a set of extensible web services that allow the rapid development of new features. In the future, LipidHome could then expand its repertoire of theoretical lipid generation rules to encompass more lipid “*Sub Classes*”, such as glycerophospholipid plasmalogens, steroids and cardiolipins, based on enhancements of the ‘FASTLipid’ library.

The strength of a sequence database or analogous resource in any “omics” field lies in the amount and detail of its annotations. These annotations enable users to search for molecules of interest and then easily digest a summary of the known properties of them and where they can easily find specific information from specialist resources. As such, LipidHome plans to incorporate the Reactome [29] knowledgebase of biological pathways in humans, to aid translating significant lipid identifications into comprehensible biological events. Bioinformatics databases rarely have static content and so to make use of the growing number of annotations and our knowledge in the field, LipidHome will re-run its annotation pipelines on a regular basis. While community agreed upon nomenclature is the basis upon which bioinformatics software is built, adoption of new standards into the wet lab and literature can be slow. To further facilitate this process, LipidHome will try to support multiple different nomenclatures and all the synonyms they provide for each lipid record. This addition will allow users to search for lipid records in nomenclatures more familiar to them, but comes with the drawback of not enforcing a standard nomenclature usage.

### Conclusion

In general, the uptake of enabling bioinformatics concepts in the field of lipidomics such as standard data formats, nomenclature, data processing and the existence of established quality control pipelines is behind that of more mature fields such as proteomics (among others). As such, there is a lot to learn by following in the footsteps of these other disciplines, by developing much needed bioinformatics resources. LipidHome represents one translation of the concept of protein sequence databases (like UniProt) to the field of lipidomics. We think that the success of lipidomics in the coming years will be closely linked to the uptake of core bioinformatics concepts and data handling best practices. LipidHome is hopefully one of a new generation of bioinformatics tools catering specifically for the lipidomics community, using modern programing approaches and long term scalable solutions to solve the challenges that are only now beginning to appear on the horizon, as the field grows. LipidHome can be accessed at http://www.ebi.ac.uk/apweiler-srv/lipidhome.

## Supporting Information

File S1 **Supplementary file containing the following.** Figure S1: The structural hierarchy of lipid identifications measurable by mass spectrometry. Figure S2: Database schema of the LipidHome database. Figure S3: The “*Category*” -> “*Main Class*” -> “*Sub Class*” hierarchy of lipids currently stored in the LipidHome database. Figure S4: Diagram that shows how lipids (e.g. glycerophospholipids) can be split in a series of building blocks. Figure S5: Screenshot of the LipidHome “Lipid record” view. Figure S6: Screenshot of the LipidHome “MS1 Search Engine”. Note S1: Details of the LipidHome annotation pipeline and its interaction with existing resources. Note S2: Details of the LipidHome web services and their usage.
(DOC)
