# Supplementary material for: LipidHome: A Database of Theoretical Lipids Optimized for High Throughput Mass Spectrometry Lipidomics
Source: PLoS One. 2013 May 7;8(5):e61951. doi: 10.1371/journal.pone.0061951 (PMC3646891; doi:10.1371/journal.pone.0061951)
Supplement: File S1 — Supplementary file containing the following. Figure S1: The structural hierarchy of lipid identifications measurable by mass spectrometry. Figure S2: Database schema of the LipidHome database. Figure S3: The “Category” -> “Main Class” -> “Sub Class” hierarchy of lipids currently stored in the LipidHome database. Figure S4: Diagram that shows how lipids (e.g. glycerophospholipids) can be split in a series of building blocks. Figure S5: Screenshot of the LipidHome “Lipid record” view. Figure S6: Screenshot of the LipidHome “MS1 Search Engine”. Note S1: Details of the LipidHome annotation pipeline and its interaction with existing resources. Note S2: Details of the LipidHome web services and their usage. (DOC) [file pone.0061951.s001.doc]

**Supplementary Figure S1:** The structural hierarchy of lipid identifications measurable by mass spectrometry. Depending on the instrumentation and downstream data analysis a variety of identification types can be detected. The lowest resolution identifications at the “*Species*” level where the total number of carbons and double bonds in the combined fatty acids is known. These are the most common identifications in high throughput studies. Higher resolution identifications may be obtained, which are capable of elucidating the individual fatty acid species (total carbons and double bonds in each chain). If the *sn* position of the chains is unresolved, this is termed a “*Fatty Acid Scan Species*” identification. Otherwise it will constitute a “*Sub Species*” identification. The highest level of structural resolution identifiable by MS approaches is the “*Isomer*” level, where fatty acid isomers are resolved (double bond positions are then known) and the *sn* positions of the fatty acid isomers are also resolved.


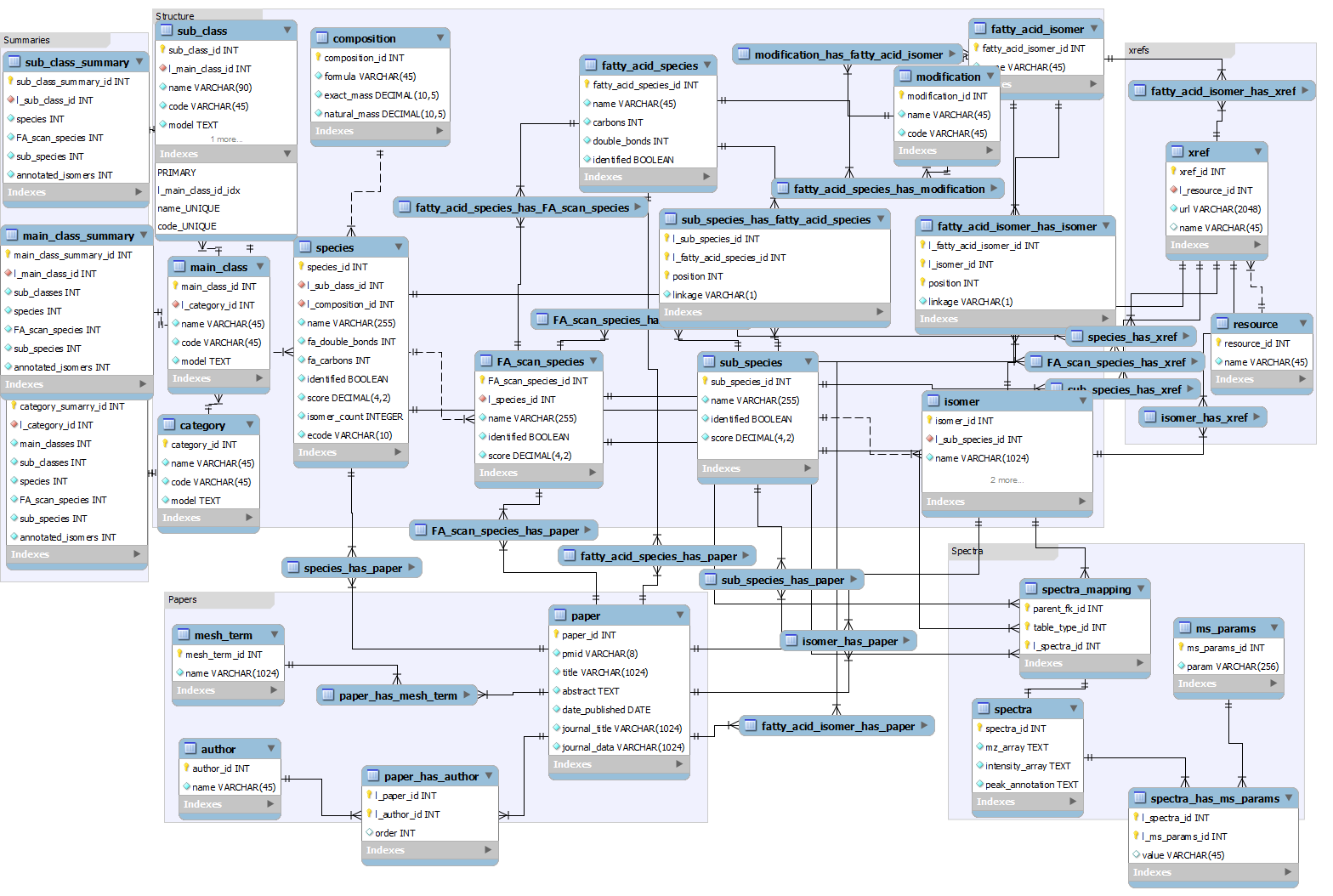


**Supplementary Figure S2:** Database schema of the LipidHome database. The schema is partitioned in five sections. The “Structure” section contains the lipid records at the various levels of the structural hierarchy. “Xrefs” contains the table which stores cross references of lipid records to external resources such as LMSD and ChEBI. “Papers” contains the abstracts, journal, title and author information of papers that mention lipid records stored in the LipidHome database in their abstracts. “Summaries” contains basic statistics on each of the classification tables that are quick to calculate once and store than recalculate upon every request. The “Spectra” set of tables is designed to house both theoretical and experimental spectra as evidence for lipid records and will be populated in a future release.

**Supplementary Figure S3:** The “*Category*” -> “*Main Class*” -> “*Sub Class*” hierarchy of lipids currently stored in the LipidHome database. “O-“ refers to an alkyl fatty acids chain linkage, this may be quantified by “d”, “t” or “e” (di, tri and tetra respectively). The prefix “L” to a “*Main Class*” code represents “Lyso” or monoacyl/monoalkyl lipid “*Sub Class*”.


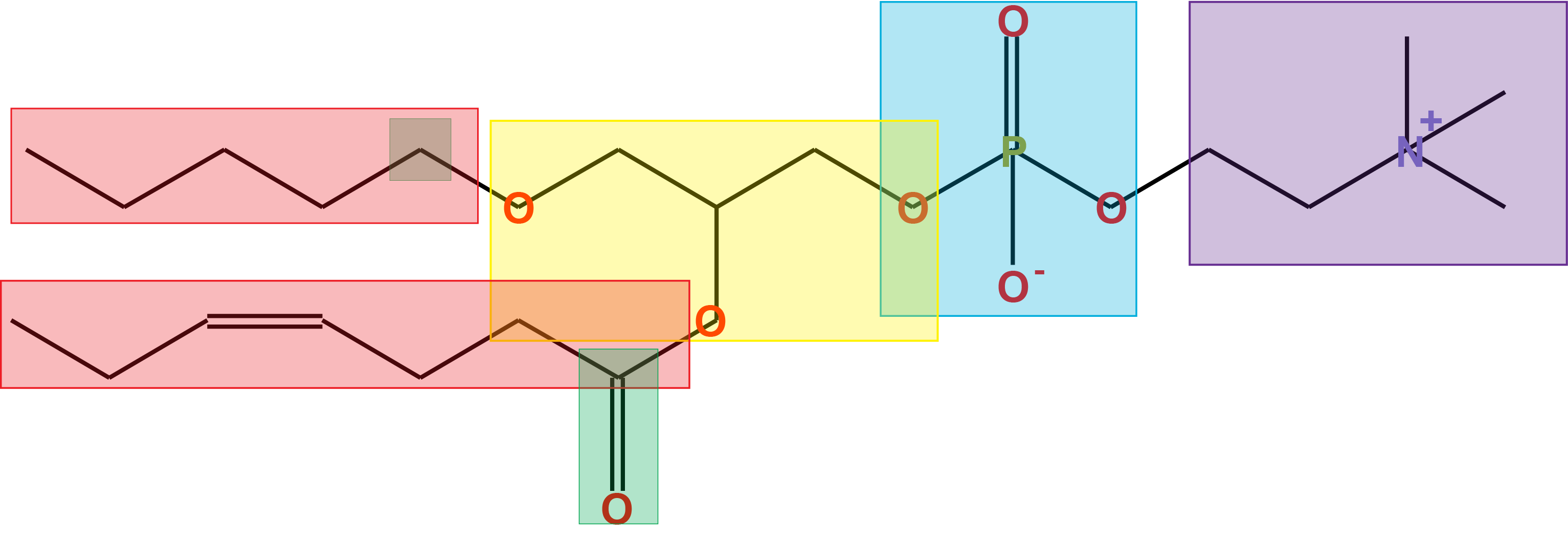


**Supplementary Figure S4:** Diagram that shows how lipids (e.g. glycerophospholipids) can be split in a series of building blocks. Red: Fatty acid with linkage. Green: Linkage. Yellow: Glycerol. Blue: Phosphate. Purple: Head group. In this case the head group is choline, but it may be substituted for a number of different molecules to create a variety of other glycerophospholipids. Breaking the molecule down into its components allows for the enumeration of all combinations of components to generate all theoretical glycerophospholipids.


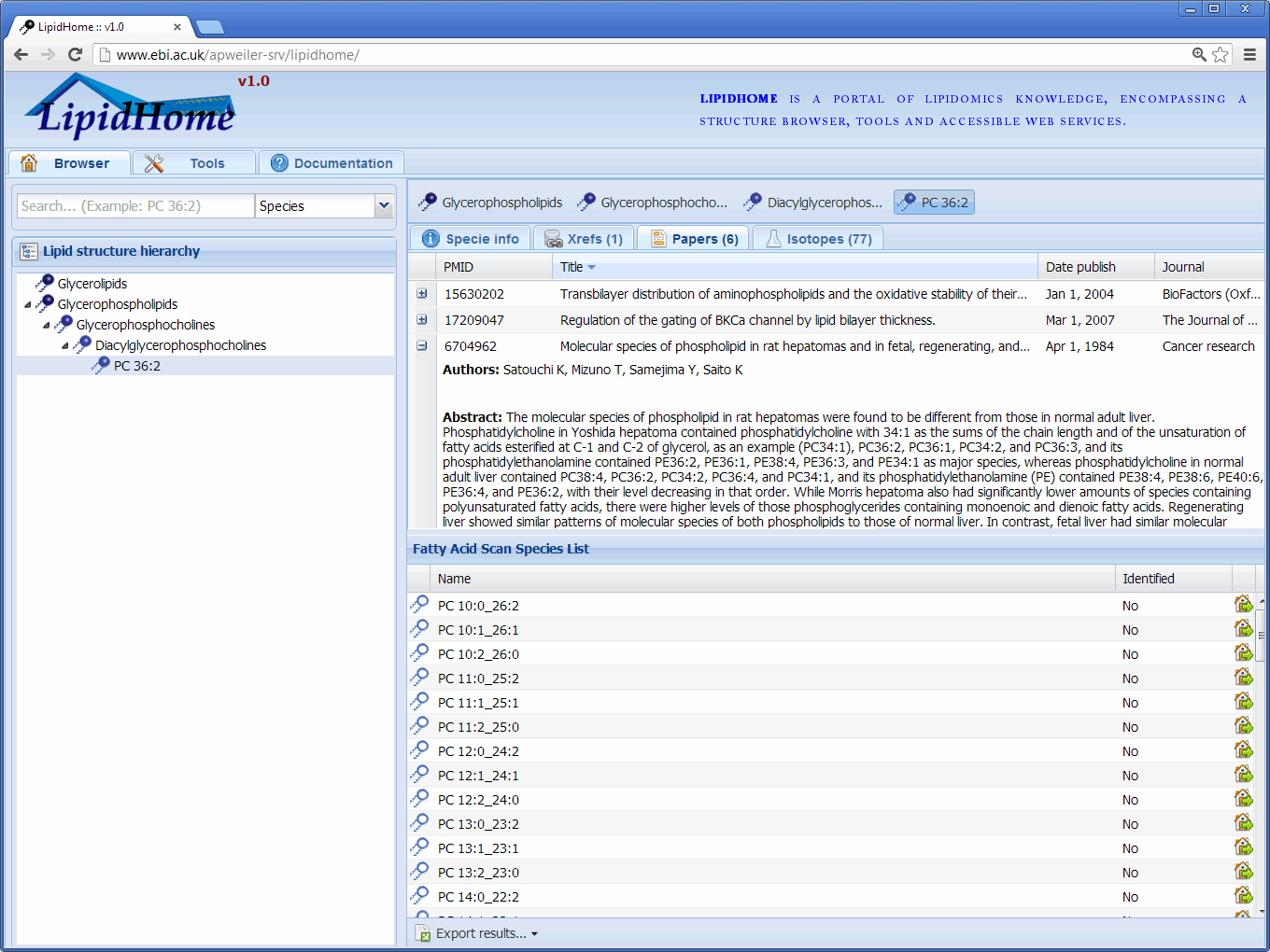


**Supplementary Figure S5:** Screenshot of the LipidHome “Lipid record” view. Alongside the general record information, a number of additional tabs that have specific information are also available, including “Xrefs” and “Papers”. Selecting the “Papers” tab shows all the abstracts in MEDLINE that contain the selected lipid record. The abstracts can be read and a link to the corresponding PubMed entry is also available.


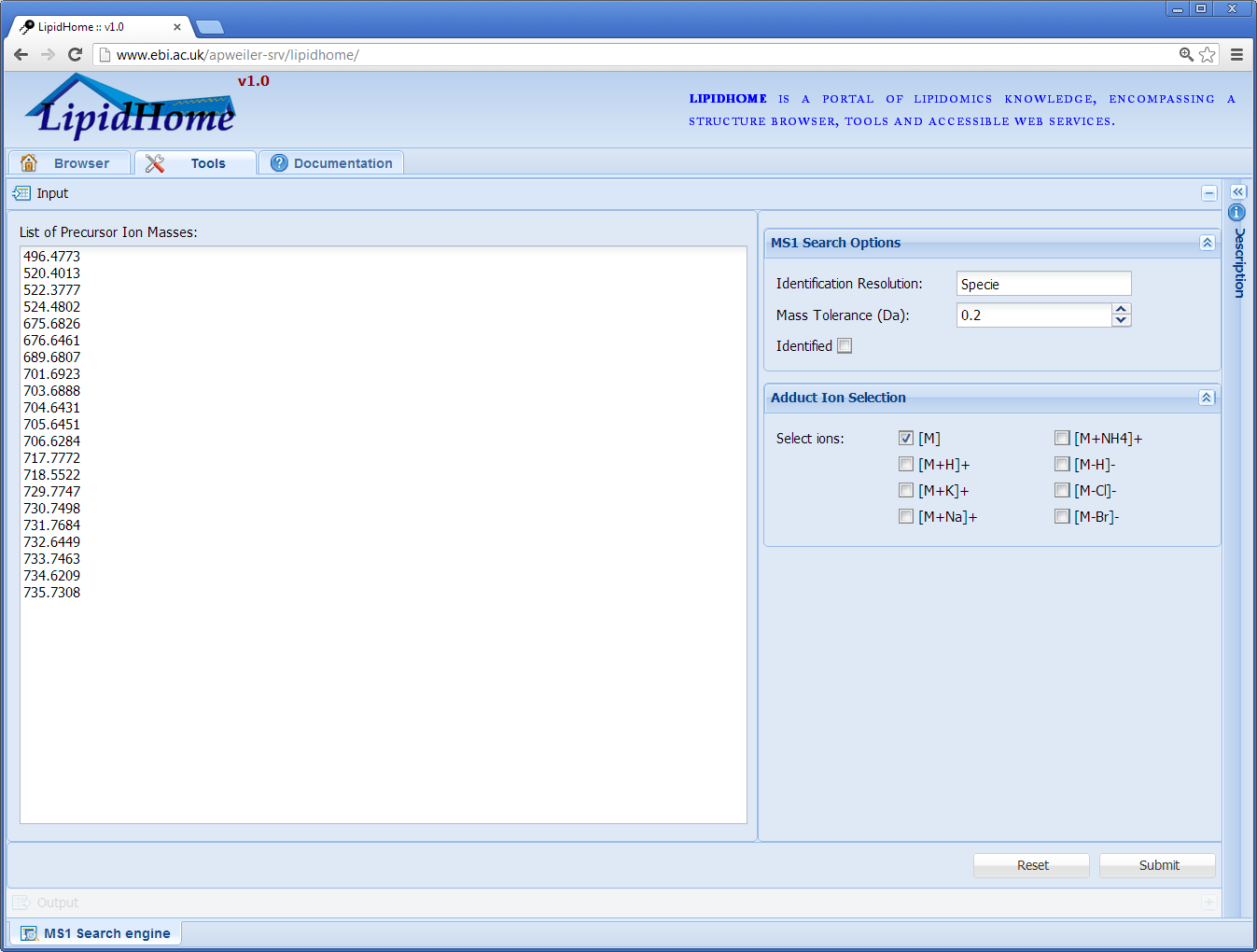


**Supplementary Figure S6:** Screenshot of the LipidHome “MS1 Search Engine”, capable of identifying a list of unknown masses with a user defined mass tolerance. Lipids classed as identified (having either a cross reference to an external resource or mentioned in an abstract in MEDLINE) can specifically be searched for. Masses can be pre-processed with a single or set of adducts ions, if the user suspects them to be present in the detected sample. Information on the parameters of the search is available by selecting the pop out “Description” panel on the far right side of the tool.

**Supplementary Note S1**

**a) LIPID MAPS**

In order to harvest cross-references from LIPID MAPS a set of scripts were written to utilise the LIPID MAPS programmatic access. The base set of “*Sub Classes*” for inclusion into LipidHome are found in LIPID MAPS and using the *LMSDSearch.php* script with the following parameters each time passing a “*Sub Class*” ID at the “?'':

http://www.lipidmaps.org/data/structure/LMSDSearch.php?Mode=ProcessTextSearch&OutputMode=File&OutputType=CSV&OutputDelimiter=semicolon&OutputQuote=no&SubClass=?

This retrieved a table of all lipids that are a member of the “*Sub Class*” in the format:

LM_ID, COMMON_NAME, SYSTEMATIC_NAME, FORMULA, MASS, CATEGORY, MAIN_CLASS, SUB_CLASS

From each of the LIPID MAPS isomer records the LipidHome parent lipids were calculated, i.e. the “*Sub Species*”, “*Fatty Acid Scan Species*” and “*Species*” it belonged to. A LipidHome “*Isomer*” was then created and persisted to the database with the appropriate links to its parent lipids. At the same time a cross reference was created and persisted for this LipidHome isomer pointing to its LIPID MAPS record ID (LMID) and URL. While the LMID is known, a LIPID MAPS PHP script was executed to retrieve the details of the record and its secondary cross references, LMID is passed into ”?'':

http://www.lipidmaps.org/data/LMSDRecord.php?Mode=File&LMID=?&OutputType=CSV&OutputDelimiter=semicolon&OutputQuote=no";

This retrieved a single semicolon delimited list of LM_ID, COMMON_NAME, SYSTEMATIC_NAME, FORMULA, MASS, CATEGORY, MAIN_CLASS,SUB_CLASS, SYNONYMS, KEGG_ID, HMDBID, CHEBI_ID,PUBCHEM_SUBSTANCE_ID, LIPIDBANK_ID, LIPIDAT_ID, STATUS,METABOLOMICS_ID. Not all LIPID MAPS records contain cross-references to every resource. Many of them point to external records at a completely different and inaccurate level of the structural hierarchy, e.g. PC 18:0/18:1[3Z] referencing the PC main class in KEGG. However, they were all harvested as it seems accepted in the field. The cross references were then persisted into the database. The pipeline is scheduled to run once a month identifying any new additions to LIPID MAPS or the secondary cross references it contains.

**b) ChEBI**

The ChEBI database of small molecules was mined for cross-references in an independent script. Using the ChEBI web service each “*Sub Species*” record in the LipidHome database was searched for an exact match in the ChEBI database. Exact matches were then persisted to the “Xrefs” section of the database.

**c) Lipid citation annotation**

Using the EBI “Whatizit” service (<http://www.ebi.ac.uk/webservices/whatizit/info.jsf>), specifically the “QBmarsdf” corpus processing pipeline, all MEDLINE abstracts were searched for each record in the database and all its synonyms. After extracting the name and ID of each record from each of the core structural tables of the database (“*Species*”, “*Fatty* *Acid* *Scan* *Species*” and “*Sub* *Species*”), the process for each record was as follows:

- The lipid names were synonymised to create a list of names that conform to a number of commonly used nomenclatures. For example the lipid “*Species*” PC 36:2 was synonymised to 36:2-PC, GPC 36:2, GlyPC 36:2 and PCho 36:2, etc.
- MEDLINE abstracts have been indexed with Lucene (an information retrieval software library, <http://lucene.apache.org/>) to allow rapid searches of large text documents. To search the indexed abstracts a Lucene search string must be created, but in order to reduce false positive search hits, the name of the “*Main* *Class*” of the record was synonymised and appended to the record name to give the search some context. For example, the “*Main* *Class*” glycerophosphocholine was synonymised to phosphatidylcholine and appended to give the two Lucene search queries ``glycerophosphocholine PC 36:2'' and ``phosphatidylcholine PC 36:2''.
- A Lucene search was performed for each Lucene search string using the EBI “Whatizit” service programmatically via the Simple Object Access Protocol (SOAP) web service. The “Qbmarsdf” pipeline was selected since it is described as a “MEDLINE Abstract Retrieval Engine based on the Text Mining Index”. It retrieved an XML document that contained a list of search results.
- The XML document was parsed to extract individual papers and their data, including PMID, title, journal, date of publication, abstract, authors and MeSH terms. In this case the paper “Molecular species of phospholipid in rat hepatomas and in fetal, regenerating, and adult rat livers. (2006). Satouchi K, Mizuno T, Samejima Y and Saito K. *Cancer Research*. 44, 1460-4. ” This information was then persisted into the appropriate tables and fields in the database referencing the relevant LipidHome record.

It took a considerable amount of time to parse all the abstracts in MEDLINE for all the synonymised records in the LipidHome database. After synonymisation the process represented millions of “Whatizit” searches and approximately 1.5 days of computation time. Due to the considerable time to perform this entire operation and the likelihood that this will increase over time as the database stores additional lipid categories, main classes and sub classes, it is scheduled to execute on the first of every month to scan for new paper annotations in the MEDLINE abstracts.

**Supplementary Note S2**

The Web services of the LipidHome web application are a simple way to computationally retrieve data from the underlying database in a standardised manner. There are several different request URLs with different parameters and return values. However, they all share in common the data interchange format JSON (JavaScript Object Notation) as the return type. It is worth noting that the web services will become more advanced and will evolve over time, as such it is worth keeping an eye on the help documentation in the main application hosted at <http://www.ebi.ac.uk/apweiler-srv/lipidhome>. All web services are available under the root path <http://www.ebi.ac.uk/apweiler-srv/lipidhome/service> and the following section's location should be appended to this root to access them.

**Category services**

Category services all relate to the retrieval of information about “*Category*” level lipids and their direct children; “*Main* *Classes*”. Available under */category* there are three methods accessible;

- */summary*

Takes a Long named `id' as parameter, this is the database id of the “*Category*” of interest. The method returns information about the specific “*Category*” such as the number of “*Main* *Classes”*, ”*Sub* *Classes*” and “*Species*” that are its members. Example: *http://www.ebi.ac.uk/apweiler-srv/lipidhome/service/category/summary?id=1*

| **Parameter** | **Values** | **Purpose** |
| --- | --- | --- |
| id | Any integer | Database record ID |

- */list*

This method takes no parameters and returns list of “*Categories*”; names and ids. The method returns a list of all “*Main* *Classes*” that are members of the selected category.

- */mainclasses*

Takes a Long named `id' as parameter, this is the database id of the “*Category*” of interest.

| **Parameter** | **Values** | **Purpose** |
| --- | --- | --- |
| id | Any integer | Database record ID |

**Main class services**

Main class services all relate to the retrieval of information about “*Main* *Class*” level lipids and their direct children; “*Sub* *Classes*”. Available under */mainclass* there are two methods accessible;

- */summary*

*Takes a Long named `id' as parameter, this is the database id of the “Main Class” of interest. The method returns information about the specific “Main Class” such as the number of “Sub Classes”, “Species” and “Sub Species” that are its members. Example:* http://www.ebi.ac.uk/apweiler-srv/lipidhome/service/mainclass/summary?id=1

| **Parameter** | **Values** | **Purpose** |
| --- | --- | --- |
| id | Any integer | Database record ID |

- */subclasses*

Takes a Long named `id' as parameter, this is the database id of the “*Main* *Class*” of interest. The method returns a list of all “*Sub* *Classes*” that are members of the selected “*Main* *class*”.

| **Parameter** | **Values** | **Purpose** |
| --- | --- | --- |
| id | Any integer | Database record ID |

**Sub class services**

Sub class services all relate to the retrieval of information about “*Sub* *Class*” level lipids and their direct children; “*Species*”. Available under */subclass* there are two methods accessible:

- */summary*

*Takes a Long named `id' as parameter, this is the database id of the “Sub Class” of interest. The method returns information about the specific “Sub Class” such as the number of “Species”, “Sub Species” and annotated “Isomers” that are its children. Example*: http://www.ebi.ac.uk/apweiler-srv/lipidhome/service/subclass/summary?id=1

| **Parameter** | **Values** | **Purpose** |
| --- | --- | --- |
| id | Any integer | Database record ID |

- */species*

Takes a Long named `id' as parameter, this is the database id of the “*Sub* *Class*” of interest. The method returns a list of all “*Species*” that are members of the selected “*Sub* *Class*”.

| **Parameter** | **Values** | **Purpose** |
| --- | --- | --- |
| id | Any integer | Database record ID |

**Species services**

Species services all relate to the retrieval of information about “*Species*” level lipids and their direct children; “*Fatty Acid Scan Species*”. Available under */specie* there are two methods accessible:

- */ summary*

*Takes a Long named `id' as parameter, this is the database id of the “Species” of interest. The method returns information about the specific “Species” such as the number of “Sub Species”, annotated “Isomers” that are its members and papers that mention it. Example*: http://www.ebi.ac.uk/apweiler-srv/lipidhome/service/specie/summary?id=1

| **Parameter** | **Values** | **Purpose** |
| --- | --- | --- |
| id | Any integer | Database record ID |

- */fascanspecies*

Takes a Long named `id' as parameter, this is the database id of the “*Species*” of interest. The method returns a list of all “*Fatty* *Acid* *Scan* *Species*” that are members of the selected “*Species*”.

| **Parameter** | **Values** | **Purpose** |
| --- | --- | --- |
| id | Any integer | Database record ID |

**Fatty acid scan species services**

Fatty acid scan species services all relate to the retrieval of information about “*Fatty* *Acid Scan Species*” level lipids and their direct children; “*Sub* *Species*”. Available under */fasscanspecie* there are two methods accessible:

- */summary*

*Takes a Long named `id' as parameter, this is the database id of the “Fatty Acid Scan Species” of interest. The method returns information about the specific “Fatty Acid Scan Species” such as the number of “Sub Species”, annotated “Isomers” that are its members and papers that mention it. Example:* http://www.ebi.ac.uk/apweiler-srv/lipidhome/service/fascanspecie/summary?id=1

| **Parameter** | **Values** | **Purpose** |
| --- | --- | --- |
| id | Any integer | Database record ID |

- */subspecies*

Takes a Long named `id' as parameter, this is the database id of the “*Fatty Acid Scan Species*” of interest. The method returns a list of all “*Sub Species*” that are members of the selected “*Fatty Acid Scan Species*”.

| **Parameter** | **Values** | **Purpose** |
| --- | --- | --- |
| id | Any integer | Database record ID |

**Sub species services**

Sub species services all relate to the retrieval of information about “*Sub Species*” level lipids and their direct children; “*Isomers*”. Available under */subspecie* there are two methods accessible:

- */summary*

*Takes a Long named `id' as parameter, this is the database id of the “Sub Species” of interest. The method returns information about the specific “Sub Species” such as the number of annotated “Isomers” that are its members and papers that mention it. Example:* http://www.ebi.ac.uk/apweiler-srv/lipidhome/service/subspecie/summary?id=1

| **Parameter** | **Values** | **Purpose** |
| --- | --- | --- |
| id | Any integer | Database record ID |

- */isomers*

Takes a Long named `id' as parameter, this is the database id of the “*Sub* *Species*” of interest. The method returns a list of all “*Isomers*” that are members of the selected “*Sub* *Species*”. These “*Isomers*” are both retrieved from the database (the identified ones harvested from other resources) and theoretically generated on the fly using the ‘FASTLipid’ Java library.

| **Parameter** | **Values** | **Purpose** |
| --- | --- | --- |
| id | Any integer | Database record ID |

**Tools services**

Tools services relate to the tools panel of the web application where currently the only application hosted is an MS1 search engine. There are two methods accessible under */tools*:

- */ms1search*

Takes several parameters and the result is a simple JSON object, each element of which is a hit against the database. This service is a POST request and must be requested as such.

| **Parameter** | **Values** |
| --- | --- |
| masses | “New line” separated list of masses |
| level | Any of “specie”, ”faScanSpecie” or “subSpecie” |
| tolerance | Any float |
| identified | Boolean |
| adductions | String of “,” separated integers representing adduct ion ids. |

- /export

The result is a downloadable file containing */ms1search* results in the specified data format.

| **Parameter** | **Values** |
| --- | --- |
| data | JSON response of /ms1search |
| format | any one of “CSV”, “TSV”, “Excel” and “XML” |

**Utility services**

Utility services is a catch all group of services necessary for varying aspects of the web application to run, most of them are application specific and superfluous to interested data consumers. However the search functionality of the LipidHome database is provided as a web service under */utils*. The result is a list of items in the database that match the search text at the level specified and some basic information about these items such as whether they are identified or not.

- */search*

Takes several parameters:

| **Parameter** | **Values** |
| --- | --- |
| query | Any String |
| type | Any of “CATEGORY”, “MAIN_CLASS”, “SUB_CLASS”, “SPECIE”, “FA_SCAN_SPECIE”, “SUB_SPECIE”, “ISOMER” AND “ALL” |
| start | Any positive integer |
| page | Any positive integer |
